# Nano-metering of Solvated Biomolecules Or Nanoparticles from Water Self-Diffusivity in Bio-inspired Nanopores

**DOI:** 10.1186/s11671-019-3178-5

**Published:** 2019-10-28

**Authors:** Luca Bergamasco, Matteo Alberghini, Matteo Fasano

**Affiliations:** 1 Department of Energy, Politecnico di Torino, Corso Duca degli Abruzzi 24, Torino, 10129 Italy; 2 Clean Water Center, Politecnico di Torino, Corso Duca degli Abruzzi 24, Torino, 10129 Italy

**Keywords:** Water quality, Emerging pollutants, Biomolecule detection, Molecular sieves, Molecular meters, Self-diffusivity

## Abstract

Taking inspiration from the structure of diatom algae frustules and motivated by the need for new detecting strategies for emerging nanopollutants in water, we analyze the potential of nanoporous silica tablets as metering devices for the concentration of biomolecules or nanoparticles in water. The concept relies on the different diffusion behavior that water molecules exhibit in bulk and nanoconfined conditions, e.g., in nanopores. In this latter situation, the self-diffusion coefficient of water reduces according to the geometry and surface properties of the pore and to the concentration of suspended biomolecules or nanoparticles in the pore, as extensively demonstrated in a previous study. Thus, for a given pore-liquid system, the self-diffusivity of water in nanopores filled with biomolecules or nanoparticles provides an indirect measure of their concentration. Using molecular dynamics and previous results from the literature, we demonstrate the correlation between the self-diffusion coefficient of water in silica nanopores and the concentration of proteins or nanoparticles contained therein. Finally, we estimate the time required for the nanoparticles to fill the nanopores, in order to assess the practical feasibility of the overall nano-metering protocol. Results show that the proposed approach may represent an alternative method for assessing the concentration of some classes of nanopollutants or biomolecules in water.

## Background

The need for water quality monitoring techniques has antique origins [1]. In ancient Rome, water sources got frequently contaminated by biological pollutants from dead bodies of men and animals or wastewater from baths, and by non-biological pollutants such as lead [2], causing severe diseases and mental problems. Continuous research and progresses across the centuries allow today most of the world population to have access to safely drinkable tap water; yet, still more than 850 million people lack primary access to clean water [3].

Accurate quality monitoring represents a challenging task, due to the different nature of the pollutants that can contaminate water, often in low concentrations. In particular, a significant number of emerging pollutants at trace levels, e.g., pharmaceuticals, chemicals, or nanomaterials, are not commonly monitored and removed by existing water treatment plants [4], although they may have adverse effects on the environment and on human health [5].

In the last decades, the advent of nanotechnologies enabled the design of tailored molecular sensors to detect different pollutants in water, such as pathogens, organic, and inorganic chemicals [6]. In their most basic version, these sensors consist of a nanostructured material, a parsing element for recognition, and an active mechanism to pass the acquired information [7]. If no quantitative information is acquired, these systems are rather referred to as nanoprobes [8] and also rely on a functionalized material to selectively detect chemicals.

In the biomedical field, the detection of biomolecules at low concentration is crucial to improve the accuracy of diagnostics and to tailor medical treatments and drugs on the needs of the patients. Ultrasensitive identification methods have been developed to this purpose, relying on a broad variety of physical and chemical phenomena, to amplify the detection signal of low-concentrated biomolecules [9–11].

In this framework, nanoporous materials have received great attention, owing to their peculiar structure, characterized by voids and channels, which makes them particularly suitable for a number of nanotechnological applications, such as catalysis [12], adsorption heat storage [13], molecular sieving [14], selective transport (membranes) [15], nanomotion [16], drug delivery [17], and biosorption [18].

Nature has greatly inspired the development of these applications, as it provides eminent examples of efficient hierarchically porous structures with specific functionalities [19, 20]. In silico optimization and properly designed synthesis allow then to overcome possible limitations, such as low stability and little resistance to harsh environments for the required applications [21, 22].

Taking inspiration from the exoskeleton (frustule) of diatom algae [23], in this work, we conceptualize nanoporous metering tablets for the concentration of some classes of biomolecules and nanoparticles in water. The key idea is to rely on the different self-diffusion coefficient that water molecules show in bulk and nanoconfined conditions, e.g., in silica nanopores. When nanoconfined indeed, water molecules have decreased mobility and thus reduced room for diffusion. The presence of a molecular solute, e.g., nanoparticles or biomolecules, further reduces the mobility depending on its size and nature, besides the size and geometry of the pore. This behavior can be accurately recovered by a scaling law previously introduced in the literature [24], therefore demonstrating that the self-diffusion coefficient of water in a nanopore allows to indirectly quantify the concentration of biomolecules or nanoparticles contained therein. Results obtained via molecular dynamics for different concentrations of proteins and iron-oxide nanoparticles in silica nanopores show that the proposed concept yields clear insight on their concentration with satisfying accuracy.

## Presentation of the Hypothesis

### Bio-inspired Nano-metering Concept

Diatoms are unicellular micro-organisms (eukaryotic algae) living in ubiquitous aqueous environments. Their cells are divided in two halves, enclosed in a silicon di-oxide shell (frustule). This porous matrix (exoskeleton) allows the living cells to interact with the external environment, optimizing the attachment of nanoparticles and active bio-molecules via the hydrophilic surface and high surface-to-volume ratio [23]. The nanopores and slits of the porous matrix, along with its chemical properties, can be used for the bioinspired design of a number of nanotechnological devices for different applications [25].

**Fig. 1:**
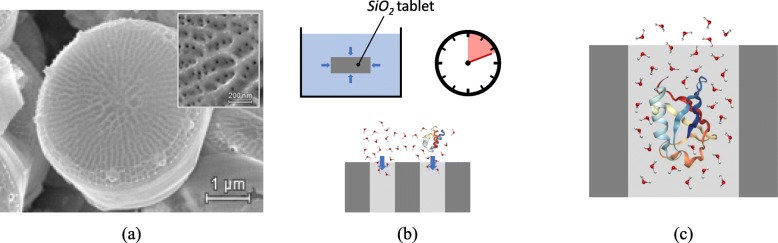
Bio-inspired conceptualization of the nano-metering silica tablets. **a** Scanning electron microscopy of *Thalassiosira pseudonana*, showing the whole valve and the detail of the porous nanochannels in the insert. Image adapted from [26] and used under CC BY 4.0 license. **b** Starting from nanopores initially empty, the water molecules and solvated proteins tend to intrude into the silica nanotablets with a characteristic filling time. **c** Detail of the intrusion of ubiquitin (1UBQ) in a silica nanopore, resulting in reduced mobility of the water molecules due to the electrostatic interactions with the protein and nanopore surfaces

Based on the structure of diatom algae, here we conceptualize nano-metering silica tablets for some classes of nanoparticles and biomolecules in water. Figure 1a shows a scanning electron microscopy of the centric diatom *Thalassiosira pseudonana* [26]. The valve consists of a porous structure characterized by channels, whose diameter ranges in the order of few nanometers, specifically around 10 nm for this case (see a detailed view in the inset). One such regular porous structure represents a natural sieve for molecules larger than the pore size, yet it allows intrusion of smaller molecules, providing a confining environment that can be exploited for our nano-metering concept.

If the diatom porous structure, or a similar one obtained via artificial synthesis [27], is immersed in a water sample with suspended pollutants, these latter are driven by capillarity and concentration gradient into the porous channels, see Fig. 1b, until equilibrium is reached. Inside the nanopores, water molecules have reduced mobility due to the nanoconfinement provided by the surface of the pore and intruded nanoparticles. As a consequence, the self-diffusion coefficient of water in the nanopore reduces with respect to that of the bulk, see Fig. 1c. The knowledge of this latter self-diffusion coefficient in the nanopore, which can be obtained, for example, via diffusion magnetic resonance imaging (D-MRI) [28, 29] or quasi-elastic neutron scattering (QENS) techniques [30, 31], allows to infer the concentration of the pollutants via the procedure explained in the next section.

### Particle Concentration from Water Self-Diffusivity

In the proximity of solid surfaces, water molecules are subject to the effect of van der Waals and Coulomb interactions; thus, they show different behavior with respect to bulk conditions [32, 33]. In particular, those solid-liquid interactions induce a layering of the water molecules close to the solid surface, which reduces their mobility and thus the self-diffusion coefficient with respect to bulk condition.

A scaling law for predicting the self-diffusion coefficient of water that encompasses this effect has been proposed in the form [24]

1 $$\begin{array}{*{20}l} D = D_{B} \left[ 1 + \left(\frac{D_{C}}{D_{B}} - 1 \right) \theta \right],  \end{array} $$

where *D*_*B*_ and *D*_*C*_ are the self-diffusion coefficients of water in bulk and fully nanoconfined conditions, respectively. In Eq. (1), *θ* is a scaling parameter that is influenced by the geometry and chemical characteristics of the solid surface, being the ratio between the nanoconfined and total water volumes in the considered configuration, namely *θ*=*V*_c_/*V*_tot_. In particular, given a certain configuration where water is nanoconfined, *V*_c_ represents the volume of water whose mobility is significantly altered by the solid-liquid interactions, and *V*_tot_ the total volume accessible by water. The nanoconfined water volume *V*_c_ can be defined as the sum of the *i*th solvent accessible surfaces *SAS*_*i*_ in the given system times an average characteristic distance $\bar {\delta }_{i}$ below which the water molecules are significantly affected by the potential well generated by the *i*th solid surface, namely:

2 $$\begin{array}{*{20}l} V_{\text{c}} = \sum_{i=1}^{N} \text{SAS}_{i} \, \bar{\delta}_{i} \,,  \end{array} $$

being *N* the number of different solid-liquid interfaces in the system. The average characteristic length of water nanoconfinement exerted by a certain solid surface $\bar {\delta }_{i}$ can be easily estimated from atomistic simulations, once the geometrical and chemical characteristics of the surface are known [24, 34]. Note that, partial overlap of the nanoconfined water volumes may occur if several solid-liquid interfaces are present, e.g., in case of a nanopore filled with nanoparticles. In this case, the scaling parameter *θ* in Eq. (1) is only apparent and may take values larger than 1, thus overestimating the actual fraction of nanoconfined water. This effect can be taken into account by continuum percolation theory (CPT) [35], which provides the effective volume fraction as [24]

3 $$\begin{array}{*{20}l} \theta^{*}=1 - \exp(-\theta).  \end{array} $$

Hence, a more accurate estimation of water nanoconfinement in case of large overlaps between confining volumes can be obtained by *θ*^∗^, which, therefore, should be better employed in Eq. (1) instead of *θ*. Clearly, *θ*^∗^≈*θ* for *θ*→0.

If the solvent accessible surface of a solvated nanoparticle (or biomolecule) is equal to *SAS*_*i*_, Eq. (2) can be used to obtain the mean nanoconfined water volume per each nanoparticle (or biomolecule) as $V_{\text {c}_{\text {i}}}={SAS}_{i} \, \bar {\delta }_{i}$. This straightforwardly yields the number of suspended nanoparticles as $\phantom {\dot {i}\!}n_{i} = V_{\text {c}} / V_{\text {c}_{\text {i}}}$ and, thus, their number concentration. Eventually, the concentration in terms of mass can be obtained via the molar mass of the considered species.

Equation 1 has been first obtained from atomistic simulations and validated against Magnetic Resonance Imaging (MRI) experiments [24]; successively, it has been validated also by QENS measures [30, 31] and applied to interpret different properties of water at solid-liquid interfaces [32, 36].

### Molecular Dynamics

Simulations are carried out to demonstrate the effectiveness of Eq. (1) to infer the nanoparticle or biomolecule concentration in a hydrated nanopore, given the self-diffusion coefficient of water therein. The open-source software GROMACS [37] is employed for the molecular dynamics (MD) simulations. To analyze the different mobility of water in bulk and nanoconfined conditions, two different geometric layouts are analyzed. For bulk conditions, a cubic computational box is adopted, where periodicity is applied along the three Cartesian axes. For nanoconfined conditions, a simplified representation of the pore in the nano-metering silica tablets is adopted, consisting of a single cylindrical nanopore (see Fig. 1c). For the sake of simplicity, but without loss of generality, a regular pore shape/size distribution is assumed, and thus, periodicity is applied along the axes.

The geometry files for the considered proteins (ubiquitin - 1UBQ; hen egg-white lysozyme - 1AKI) were obtained from the Protein Data Bank [38] database, whereas both the silica nanopores and magnetite nanoparticles were available from a previous study [24]. The intra-molecular bonded interactions in the silica nanopores and iron oxide nanoparticles are modeled by harmonic stretching and angle potentials, as detailed in [24]. Their non-bonded interactions are modeled by 12-6 Lennard-Jones and electrostatic potentials, as also reported in [24]. Bonded and non-bonded interactions of the proteins are taken from GROMOS96 43a2 [39]. Notice that, during the equilibration, all bonds in the proteins are kept rigid using the LINCS (Linear Constraint Solver) algorithm [40]. The SPC/E water model [41] with rigid bonded interactions is adopted in all cases, as it accurately recovers the most relevant properties of water at room temperature [42].

In both bulk and nanoconfined configurations, the system is first energy minimized, solvated (water density approximately equal to 1.00 g/cm^3^) and, for proteins, the net charge neutralized via ion addition. In detail, chloride ions are introduced in the simulation box to neutralize the net positive charge of lysozyme, whereas ubiquitin is neutral and thus does not require any ion addition. Next, the hydrated system is relaxed to its energy minimum for a sufficient time. The temperature of the system is then equilibrated by a simulation in the NVT ensemble for 100 ps (*T*=300 K, Nosé-Hoover thermostat) to achieve convergence of potential energy in the simulated configuration (about ± 1% fluctuations around the equilibrium value, see Additional file 1: Figure S1c). After that, bulk configurations are also equilibrated in the NPT ensemble for 100 ps (*T*=300 K, Nosé-Hoover thermostat; *p* = 1 bar, Parrinello-Rahman barostat) to achieve convergence of water density in the simulated setups (about ± 2% fluctuations around the equilibrium value, see Additional file 1: Figure S1a). The production run is finally performed in the NVT ensemble (*T*=300 K, Nosé-Hoover thermostat). In all simulated cases, steady state is considered as reached when the self-diffusion coefficient, which is evaluated every 100 ps, tends to an asymptotic value (i.e., ± 10% fluctuations around the moving average, see Additional file 1: Figure S1b and d). Since this is generally achieved after ≈ 500 ps for the bulk configurations or ≈ 1000 ps for the nanoconfined ones, the former are continued up to 1 ns, the latter up to 2 ns to have better statistics. In all runs, the leap-frog algorithm with time step 0.001 ps is used, while a 1.2-nm cut-off distance is adopted for the van der Waals interactions and a Particle Mesh Ewald (PME) method for the electrostatic ones (mesh spacing 0.16 nm). The solvent accessible surface of the solid nano-objects is obtained from the production run and fed into a dedicated routine (see the Supplementary software in [24]), which, based on the adopted force field, computes the average characteristic length of nanoconfinement $\bar {\delta }_{i}$ per each *i*th solid-liquid interface in the setup.

## Testing the Hypothesis

### Water Self-Diffusion for Different Systems

The validity of the scaling law in Eq. (1) has been first tested considering both results from the literature (14 configurations) and new simulations (9 configurations). In particular, the configurations taken from the literature are hydrated silica nanopores with diameter *d*_*P*_=8.13 or 11.04 nm (see Supplementary Table S1 in [24]); sole magnetite nanoparticles with diameter *d*_*p*_=1.27 or 1.97 nm immersed in cubic water boxes with 6 or 7 nm side, respectively (see Supplementary Table S4 in [24]); sole 1AKI or 1UBQ proteins immersed in cubic water boxes with 7.03 or 6.32 nm side, respectively (see Supplementary Table S10 in [24]); a hydrated silica nanopore with diameter *d*_*P*_=8.13 nm filled with 2, 4, 8, or 16 magnetite nanoparticles with diameter *d*_*p*_=1.97 nm (see Supplementary Table S2 in [24]) or 16 magnetite nanoparticles with diameter *d*_*p*_=1.27 nm (see Supplementary Table S3 in [24]); and a hydrated silica nanopore with diameter *d*_*P*_=11.04 nm filled with 36 or 66 magnetite nanoparticles with diameter *d*_*p*_=1.27 nm or 20 magnetite nanoparticles with diameter *d*_*p*_=1.97 nm (see Supplementary Table S3 in [24]). Furthermore, the new simulated setups are a hydrated silica nanopore with diameter *d*_*P*_=8.13 nm filled with one 1UBQ protein and a hydrated silica nanopore with diameter *d*_*P*_=11.04 nm filled with 2, 3 or 9 1AKI proteins, or 2, 7, 9 or 12 1UBQ proteins.

**Fig. 2:**
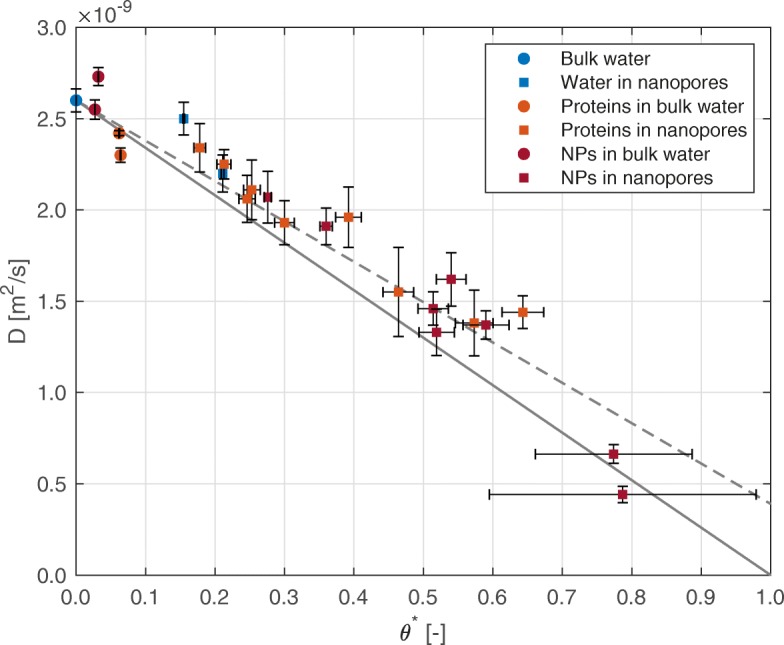
Water self-diffusion coefficient for different systems. The self-diffusion coefficient of water is shown against the scaling parameter *θ*^∗^ for different systems. Data for proteins in silica nanopores have been obtained via molecular dynamics, while the rest of the data from the Supplementary information in [24]. In the legend, nanoparticles are abbreviated as NPs. The uncertainties on the value of *D* refer to the fitting of the mean square displacement (±1 s.d.); the uncertainties on the value of *θ*^∗^ to the estimate of the total volume accessible to water molecules (±1 s.d.). The solid and dashed lines report the model in Eq. (1) in case of *D*_*C*_=0 and *D*_*C*_=0.39×10^−9^m^2^/s, respectively

Figure 2 shows the scaling behavior for the self-diffusion coefficient of water for the different systems previously listed, namely bulk water (*D*_*B*_=2.60×10^−9^m^2^/s), hydrated silica nanopores, solvated proteins and magnetite nanoparticles, and hydrated silica nanopores filled with proteins or nanoparticles. As expected, water inside the silica nanopores shows reduced self-diffusion, coherently with the increasing degree of nanoconfinement represented by the scaling parameter *θ*^∗^. Suspended molecules (nanoparticles and proteins) show a similar effect on the self-diffusion coefficient of water. In Fig. 2, the solid line corresponds to Eq. (1) with *D*_*C*_/*D*_*B*_≈0, which represents the limiting case of assuming that nanoconfined water molecules have no mobility and are therefore unable to diffuse. The dashed line corresponds, instead, to the same equation with a more realistic value of *D*_*C*_=0.39×10^−9^m^2^/s, as observed in the simulations reported in [24]: this model is able to accurately recover the simulation results (*R*^2^= 0.93), therefore confirming the good prediction capabilities of Eq. (1) also for the new simulated configurations.

### Examples of the Proposed Protocol in Practice

Let us consider a nanoporous silica tablet used as metering device for a known polluting agent in water, as proposed. Let us assume that the tablet is left immersed in a solution test sample for sufficient time so that the suspended polluting molecules diffuse into the tablet and equilibrium is reached (see the next section for a detailed discussion on this). The sample is then extracted and the self-diffusion coefficient of water *D* inside the porous structure of the tablet obtained by, e.g., QENS measure. The volume fraction *θ*^∗^ can be then easily obtained from Eq. (1), since both *D*_*B*_ and *D*_*C*_ are known at a given temperature. Then, the overlapped nanoconfined volumes of water can be taken into account by CPT, thus leading to *θ*=− ln(1−*θ*^∗^). For a single type of polluting agent enclosed in one nanopore, Eq. (2) simplifies to

4 $$\begin{array}{*{20}l} V_{\text{c}} = n_{p} \text{SAS}_{p} \, \bar{\delta}_{p} + \text{SAS}_{P} \, \bar{\delta}_{P} \,,  \end{array} $$

being the subscripts *p* and *P* referred to the particles and pore, respectively. Once the solvent accessible area *SAS* and the mean characteristic length of nanoconfinement $\bar {\delta }$ for particles and pores are known from molecular dynamics, the number of suspended particles is easily obtained as

5 $$\begin{array}{*{20}l} n_{p} = \frac{V_{\text{tot}}\theta-\text{SAS}_{P} \bar{\delta}_{P}}{\text{SAS}_{p} \bar{\delta}_{p}}.  \end{array} $$

**Fig. 3:**
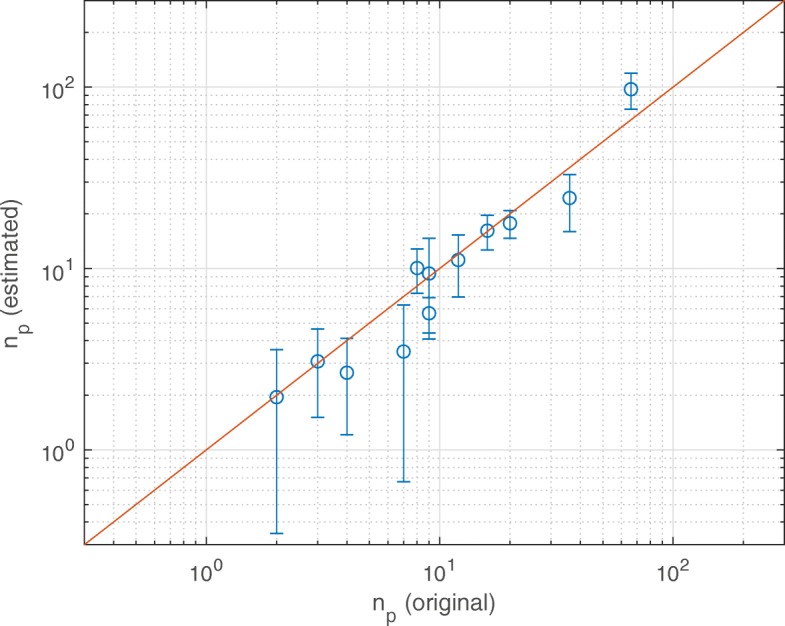
Accuracy of the proposed nano-metering protocol. Estimated number of particles using the proposed protocol vs the original number of particles. The reported data (dots) refer to the configurations in Table 1; the solid line is the bisector. The error bars on the value of $n_{p}^{e}$ are computed from the variability of *D* and *V*_tot_ values (uncertainty quantification, ± 1 s.d.)

The results of this nano-metering procedure are reported in Tab. 1 and Fig. 3, for some sample cases of proteins and nanoparticles inside silica nanopores from Fig. 2. In particular, the bisector curve in Fig. 3 allows to appreciate the accuracy of the estimated number of suspended particles $(n_{p}^{e})$ with respect to the original (actual) one $(n_{p}^{o})$, being the *R*^2^ of the curve equal to 0.85.

**Table 1:** Data employed for demonstrating the effectiveness of the proposed nano-metering protocol

MOL	*d*_*P*_[nm]	*d*_*p*_[nm]	$n_{p}^{o}$	*D* [*m*^2^/s]	*θ* ^∗^	*θ*	*V*_tot_[n*m*^3^]	*δ*_*p*_[nm]	SAS_*p*_[n*m*^2^]	*δ*_*P*_[nm]	SAS_*P*_[n*m*^2^]	$n_{p}^{e}$
IONP	8.13	1.97	4	1.91E–09	0.31	0.37	200.75	0.50	21.64	0.33	140.47	2.66
IONP	8.13	1.97	8	1.33E–09	0.57	0.85	180.90	0.50	21.64	0.33	140.47	10.06
IONP	8.13	1.27	16	1.46E–09	0.52	0.73	193.59	0.46	12.61	0.33	140.47	16.16
IONP	11.04	1.27	36	1.62E–09	0.44	0.59	351.63	0.46	12.61	0.33	194.00	24.47
IONP	11.04	1.97	20	1.37E–09	0.56	0.81	313.15	0.50	21.64	0.33	194.00	17.74
IONP	11.04	1.27	66	6.63E–10	0.88	2.09	300.98	0.46	12.61	0.33	194.00	97.30
1AKI	11.04	–	2	2.06E–09	0.24	0.28	375.71	0.32	67.36	0.33	194.00	1.95
1AKI	11.04	–	3	1.93E–09	0.30	0.36	357.35	0.32	67.36	0.33	194.00	3.08
1AKI	11.04	–	9	1.44E–09	0.52	0.74	247.23	0.32	67.36	0.33	194.00	5.66
1UBQ	11.04	–	7	1.96E–09	0.29	0.34	338.10	0.31	48.19	0.33	194.00	3.48
1UBQ	11.04	–	9	1.55E–09	0.47	0.64	316.87	0.31	48.19	0.33	194.00	9.38
1UBQ	11.04	–	12	1.38E–09	0.55	0.81	285.02	0.31	48.19	0.33	194.00	11.14

Data for the iron-oxide nanoparticles (IONP) have been taken from the supplementary information in [24], while those for proteins (1AKI and 1UBQ) have been computed for the present work. Notice that Eq. (1) has been applied considering *D*_*C*_=0.39×10^−9^m^2^/s. $n_{p}^{e}$ is the number of particles estimated using the proposed protocol; $n_{p}^{o}$ the original (actual) number of particles in the simulated setups

Given the number of biomolecules or nanoparticles in the nanopore, their number concentration can be readily obtained as *c*=*n*_*p*_/*V*_P_, being $V_{\text {P}}= T \pi d_{P}^{2}/4$ the free volume of the pore in case of a cylindrical through configuration [27] (*T* is the pore length, that is, e.g., the thickness of the silica tablet in case of straight pores). This nano-metering protocol has been here presented for a single pore, but it could be easily extrapolated to the whole nano-metering tablet given its porosity and thus number of hydrated nanopores.

### Filling of the Nanopores

The examples discussed in the previous section account for equilibrium conditions, thus assuming that the concentration of particles in the nanopore equals that in the bulk solution. Nevertheless, the nano-metering protocol suggested in this work would also involve the filling process of nanopores by the solvated particles to be detected. In this section, we assess the practical feasibility of the suggested nano-metering protocol with respect to the characteristic filling time of the nanopores.

The experimental protocol commonly used to maximize the filling of nanopores by solvated nanoparticles includes sonication and centrifugation processes [43], which in some cases may lead to a non-uniform distribution of particles due to cluster creation and clogging of the nanochannels [44–47]. Here, we consider a spontaneous solvent imbibition and diffusion of the dispersed particles into the initially dry nanopores. Hence, we adopt a simplified approach, considering two successive processes because of the very different time scales of the involved phenomena: capillary imbibition of the dry pores by the pure fluid, and particle diffusion by Fickian mechanism through the hydrated pores to equilibrium conditions.

Experiments and molecular dynamic simulations [48–50] show that if the average capillary diameter is greater than approximately four times the molecular diameter of water [50, 51], the imbibition process can be described by the Lucas-Washburn (LW) equation. Under the sharp-front approximation, Darcy’s law can be used to model the position of the moving front *h*(*t*), recovering the same form of the LW equation [52]:

6 $$  h=\sqrt{\frac{2K \Delta p}{\phi_{i} \mu}t},  $$

where *Δp* is the driving capillary pressure, *μ* is the dynamic viscosity of the fluid (water in this case), *ϕ*_*i*_ is the effective porosity of the medium at the beginning of the uptake process, and *K* is its permeability. Porous silica materials present a very regular structure and a narrow pore size distribution [46]; thus, their permeability can be computed as [49, 53]:

7 $$  K=\frac{1}{8}\frac{r_{h}^{4}\phi_{0} }{r_{0}^{2} \tau },  $$

where *r*_0_ is the nominal pore diameter, *r*_*h*_ is the hydraulic diameter of the pore (smaller than *r*_0_ because of the adsorbed layer of water molecules on the capillary surface), *ϕ*_0_ is the nominal porosity of the medium, and *τ* is its tortuosity. The capillary pressure can be described by the Young-Laplace equation:

8 $$  \Delta p=\frac{2\sigma \cos (\vartheta)}{r_{h}},  $$

where *σ* is the surface tension of the fluid and *𝜗* its dynamic contact angle with respect to the pore surface. Note that, for silica-water interfaces, *𝜗*≈0 [49, 54].

**Fig. 4:**
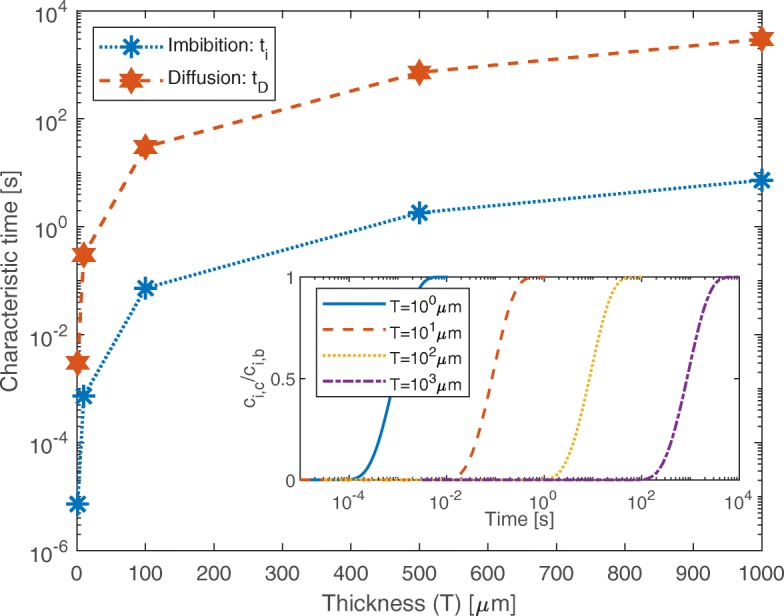
Characteristic filling times of the nanopores. Characteristic times of the nanopore filling by capillarity (blue dotted line, *t*_*i*_) and particle diffusion (red dashed line, *t*_*D*_) varying the thickness of the nanoporous silica tablet (nanopores with 11.04-nm diameter; solution of 1AKI proteins in water at 1% weight fraction). The particle diffusion process through the fully hydrated pores requires a characteristic time *t*_*D*_ two orders of magnitude higher than *t*_*i*_, in all the analyzed configurations. The inset shows the ratio between the particle concentration at the center of the nanopore (*c*_*i*,*c*_, at *x*=*T*/2) and the bulk one (*c*_*i*,*b*_, at *x*=0 and *T*) as a function of time for different thickness (*T*) of the tablets

Nanoporous tablets can be precisely manufactured with straight cylindrical pores ranging from 5 to 150-nm diameter and porosity from 40 to 90% [27]. Equation 6 can be employed to estimate the time required for the complete imbibition of a nanoporous material with such geometrical characteristics (*t*_*i*_), in the simplifying hypothesis that diluted contaminants do not affect this process. The resulting *t*_*i*_ are reported in Fig. 4 using blue asterisks and dotted line, for tablet thickness (i.e., pore length) varying from 1 *μ*m to 1 mm. The results show a remarkable speed of the imbibition process: the thickest macroscopic tablet considered is completely filled with water in less than 10 s. In order to analyze coherent configurations with those simulated by molecular dynamics setups, the estimates of *t*_*i*_ consider an initially dry material (i.e., *ϕ*_0_=*ϕ*_*i*_), an average pore diameter of *d*_0_=2*r*_0_=11.04 nm, porosity and tortuosity equal to *ϕ*_0_≈40*%* and *τ*≈1, respectively. Since the hydrodynamic radius should take into account the effect of adsorbed water molecules, *r*_*h*_=*r*_0_−2*d*_*w*_, where two layers of adsorbed water molecules (with *d*_*w*_=0.275-nm diameter) are assumed [24]. The remaining water within the pores can be reasonably considered at bulk conditions, and thus, *σ*=0.072 N/m and *μ*=10^−3^ Pa ·s at *T*=300 K. These estimates are performed neglecting the effect of the suspended particles on the imbibition process. However, particle-wall interactions are not negligible at high volume fractions or for particle-pore size ratios close to unit, as local properties of water such as viscosity and contact angle may be altered [55]. Still, the position of the liquid front can be described by Eq. (6) for particle-pore size ratios of 10% or lower [55].

The characteristic time required for particle diffusion into the fully hydrated, homogeneous, and straight nanopores is then assessed. This filling process is assumed to rely on pure diffusion [56, 57] and, under the assumption of negligible particle-pore interactions, can be described by Fick’s equation:

9 $$  \phi_{0}\frac{\partial c_{i}}{\partial t}-D_{e}\nabla^{2} c_{i}=0,  $$

being *c*_*i*_ the particle concentration, *D*_*e*_=*ϕ*_0_*D*_*p*_/*τ* the effective diffusivity of the particles in the hydrated channels, and *D*_*p*_ their diffusivity in the bulk fluid.

Computations are performed, as a first example, considering a diluted lysozyme (1AKI) solution at *c*_*i*,*b*_= 3.4 mol/*m*^3^ concentration, i.e., approximately 1% weight fraction. Similarly to the configuration employed to estimate *t*_*i*_, silica tablets with an average pore diameter of *d*_0_=2*r*_0_=11.04 nm, varying thickness, porosity equal to *ϕ*_0_≈40*%*, and tortuosity *τ*≈1 are considered. Starting from a fully hydrated pore without any particle inside, the filling time *t*_*D*_ is estimated as the time required to reach *c*_*i*,*c*_=0.95*c*_*i*,*b*_ at the center of the pore, namely at *x*=*T*/2. The particle concentration is constant and equal to *c*_*i*,*b*_ at both ends of the channel, namely at *x*=0 and *T*. The diffusion coefficient of the lysozyme in water is assumed equal to the bulk value, namely *D*_*p*_=11.08·10^−11^ *m*^2^/s [58, 59]. Equation 9 is solved numerically in one dimension by a finite-element method. The results are reported in Fig. 4 as red stars and dashed line, showing that *t*_*D*_ is about two orders of magnitude higher than *t*_*i*_ for a given thickness of the silica tablet. Even in the worst case presented (*T*=1 mm, *t*_*D*_≈3000 s), the filling time appears to be compatible with a nano-metering protocol of practical interest. Note that both simulations [60] and experiments [61] in the literature show that the particle diffusivity *D*_*p*_ in nanopores can be significantly lower than the bulk one, because of the different affinity of particles with the pore surface and the presence of nanoconfined water with low mobility. Hence, the proposed approach provides initial indications on the characteristic filling time but, to achieve more accurate estimations, *D*_*p*_ and thus *t*_*D*_ should be analyzed on a case-by-case basis [62].

As a second example, we assess the possibility of metering the concentration of solvated drugs, since they are currently considered as emerging pollutants of water sources [5]. In particular, we analyze one of the relevant drugs for cancer treatment: doxorubicin, which is a hydrophobic molecule commonly used for chemotherapy [63–65]. An estimation of the diffusion time *t*_*D*_ of doxorubicin into the hydrated silica nanotablets can be performed under the assumptions already adopted for the previous case study. Unbound doxorubicin has a diffusion coefficient of *D*_*p*_=1.6·10^−10^ *m*^2^/s [66]; thus, a silica tablet with 500 *μ*m thickness would be filled at 95% of the bulk concentration (*c*_*i*,*b*_= 3.4 mol/*m*^3^) in approximately 500 s. This illustrative case shows that the proposed nano-metering protocol could be also potentially employed to detect the concentration of drug traces in water. We remark that the effect of additional factors (e.g., chemical affinity between drugs and pore surface, pH, presence of surfactants or functionalizations), which are not considered in this simplified model, should be experimentally investigated, as they may significantly deviate the characteristic time with respect to the considered simplified conditions.

Clearly, the filling time of the nanopores should lie between *t*_*i*_ (best case, nanoparticles are dragged into the pores together with water by capillarity) and *t*_*D*_ (worst case, water first hydrates the pores and then nanoparticles follow by Fickian diffusion). Even in the worst explored case, modeling estimations of the filling time of the nanopores indicate a practical feasibility of the proposed nano-metering protocol. This idea is also supported by some promising experimental evidences in the literature. For instance, hydrophilic carbon nanotubes with average diameter of 300 nm are easily filled by spontaneous imbibition with particles in the range of 10–50 nm [67, 68], proving that a proper tuning of the geometrical and chemical parameters of the configuration would provide a fast and homogeneous filling of the nanochannels, thus making the proposed nano-metering protocol feasible.

## Implications of the Hypothesis

Inspired by the regular nanoporous structure of diatom algae frustules, in this work, we have presented a new concept for measuring the concentration of nanoparticles or biomolecules dispersed in water. The regular structure of the algae frustules can be artificially reproduced by nanoporous silica tablets, whose pore size, thickness, and shape should be precisely tuned to optimize the selective uptake of particles. The proposed nano-metering method relies on the effect of those nanoparticles or biomolecules on the self-diffusion coefficient of water nanoconfined within the tablet’s pores, and consists in the following steps:

1. Synthesize porous tablet with a controlled size distribution of nanopores.
2. Let the nanopores of the tablet fill with the solution containing the particles to be detected via capillary imbibition and particle diffusion, achieving equilibrium conditions between the nanopores and the surrounding solution.
3. Remove the tablet from the solution and measure the self-diffusion coefficient of water in the hydrated nanopores filled with the particles, e.g., by QENS or D-MRI techniques.
4. Correlate the measured self-diffusion coefficient of water with the particle concentration by means of Eqs. 1 to (5). The solvent accessible surface of nanopore and particles (*SAS*) and their mean characteristic length of nanoconfinement ($\bar {\delta }$) should be computed from molecular dynamics or taken from available databases.

Molecular dynamic simulations and evidence from the literature have been employed to assess the feasibility of the proposed nano-metering protocol. Hydrated nanopores filled with different concentrations of iron-oxide nanoparticles or proteins have been analyzed, finding agreement between the computed and predicted self-diffusion coefficient of nanoconfined water, thus allowing to estimate the particle concentration. A preliminary analysis of the mechanisms involved in the nanopores filling has been also carried out. Because of the different time scales, two different phenomena have been considered separately: the imbibition of a dry tablet by pure water, driven by capillarity, and the particle diffusion through the hydrated pores, driven by concentration gradient. Results show that the leading characteristic time in the filling process is the time required for particles to diffuse into the hydrated pores; however, the estimated filling time does not exceed 1 h even in case of the thickest tablets considered (1 mm), therefore not compromising the practical feasibility of the nano-metering protocol.

Although the proposed nano-metering method has shown promising results from a numerical point of view, the actual experimental implementation may have to face some additional issues. First, the interaction between the pore surface and particles could be non-negligible and thus alter the filling process (e.g., pores clogging). This effect could generate a bias between the actual concentration of the particles in the bulk solution and the one measured within the pores. Such an issue could be solved by an accurate selection of the surface properties of the pores, which should not interact with the particles to be detected. Second, the current experimental techniques could have difficulty to measure the water diffusivity with a single-nanopore resolution. This issue could be mitigated by measuring the average self-diffusion coefficient over hundreds or thousands of nanopores, which could also provide a better statistical sampling in case of inhomogeneous particle filling throughout the tablet. Third, the uncertainty of the nano-metering protocol should be assessed by experiments. The configurations studied by molecular dynamics have revealed prediction errors up to ± 50*%*: this error range could be eventually reduced by considering larger statistical samples, both in terms of time (multiple measures) and space (averages over hundreds or thousands of pores). Fourth, the optimal diameter of the nanopores should be determined on the basis of the expected size and concentration of the particles to be detected. On the one hand, the pore size should be chosen to avoid low *θ*^∗^ (e.g., *θ*^∗^ should be > 0.2), since this could lead to negligible variations of the self-diffusivity of water that could be eventually below the resolution of the QENS or D-MRI techniques; on the other hand, high levels of water nanoconfinement should be avoided as well (e.g., *θ*^∗^ should be < 0.8), to limit the risk of pore clogging or particle aggregation/segregation and thus biased concentration results.

In conclusion, further research is needed to validate experimentally the original nano-metering protocol discussed in this work. However, the presented numerical results prove the potential of the idea, which may pave the way to a completely new class of detection processes of emerging nanopollutants in water or biomolecules. In perspective, the microscopic size of the metering devices, e.g., nanoporous silica tablets, may allow automation of the nano-metering process through lab-on-a-chip devices.

## Supplementary information

**Additional file 1** PDF file containing a multi-panel figure showing the energy, density, and self-diffusion coefficient convergence for some illustrative molecular dynamics simulations (bulk and nanoconfined setups)

## Data Availability

The datasets used and/or analyzed during the current study are available from the corresponding author on reasonable request.

## Abbreviations

1AKI: Lysozyme
1UBQ: Ubiquitin
CPT: Continuum percolation theory
D-MRI: Diffusion magnetic resonance imaging
IONP: Iron oxide nanoparticle
LINCS: Linear constraint solver
LW: Lucas-Washburn
MD: Molecular dynamics
MOL: Molecule
MRI: Magnetic resonance imaging
NP: Nanoparticle
PME: Particle Mesh Ewald
QENS: Quasi-elastic neutron scattering
SAS: Solvent accessible surface
