# Supplementary material for: Nano-metering of Solvated Biomolecules Or Nanoparticles from Water Self-Diffusivity in Bio-inspired Nanopores
Source: Nanoscale Res Lett. 2019 Oct 28;14:336. doi: 10.1186/s11671-019-3178-5 (PMC6816642; doi:10.1186/s11671-019-3178-5)
Supplement: Supplementary file 1 — Additional file 1 PDF file containing a multi-panel figure showing the energy, density, and self-diffusion coefficient convergence for some illustrative molecular dynamics simulations (bulk and nanoconfined setups) [file 11671_2019_3178_MOESM1_ESM.pdf]

NANO IDEA

Nano-metering of solvated biomolecules or nanoparticles from water self-diffusivity in bio-inspired nanopores

Luca Bergamasco[1], Matteo Alberghini[1,2] and Matteo Fasano[1]

Supplementary information

Convergence of the atomistic simulations

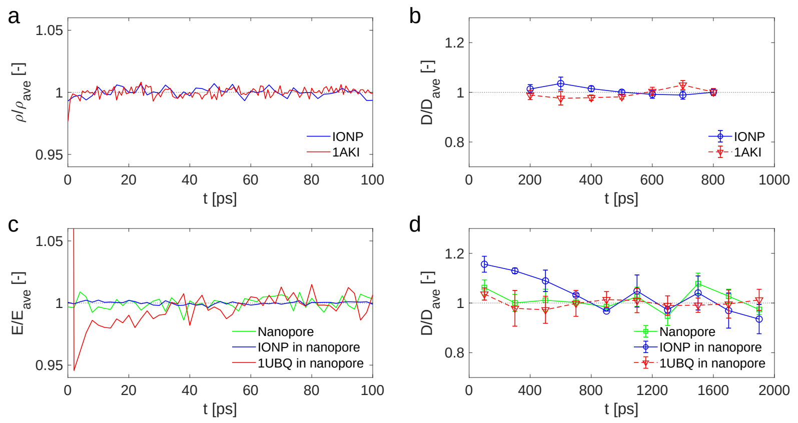

Figure S1 Convergence of the equilibration and production runs for some of the simulated configurations. (a) Convergence of water density (ρ) to the bulk value at 300 K ($\rho_{ave} \approx 1$ g cm^{-3}) during NPT equilibration. The blue line refers to the configuration where the iron oxide nanoparticle (IONP) with 1.27 nm diameter is solvated in a cubic box of water with 6 nm side; the red line to the lysozyme (1AKI) in a cubic box of water with 7.03 nm side. (b) Convergence of the self-diffusion coefficient of water (D) to an equilibrium value (D_{ave}, obtained from the moving average) during the production run. The blue line refers to the configuration where the IONP with 1.27 nm diameter is solvated in a cubic box of water with 6 nm side; the red line to the 1AKI in a cubic box of water with 7.03 nm side. (c) Convergence of potential energy (E) to an equilibrium value (E_{ave}) during NVT equilibration. The green line refers to the configuration where the silica nanopore with 8.13 nm diameter is hydrated by water; the blue line to 8 IONPs with 1.97 nm diameter within the hydrated silica nanopore with 8.13 nm diameter; the red line to 7 ubiquitins (1UBQ) within the hydrated silica nanopore with 11.04 nm diameter. (d) Convergence of D to an equilibrium value D_{ave} during the production run. The green line refers to the configuration where the silica nanopore with 8.13 nm diameter is hydrated by water; the blue line to 8 IONPs with 1.97 nm diameter within the hydrated silica nanopore with 8.13 nm diameter; the red line to 7 1UBQ within the hydrated silica nanopore with 11.04 nm diameter. Error bars refer to ± 1 standard deviation.

[1] Department of Energy, Politecnico di Torino, Turin, Italy
[2] Clean Water Center, Politecnico di Torino, Turin, Italy

Full author information is available at the end of the main article.
